# The IMMENSE Study: The Interplay Between iMMune and ENdothelial Cells in Mediating Cardiovascular Risk in Systemic Lupus Erythematosus

**DOI:** 10.3389/fimmu.2020.572876

**Published:** 2020-10-29

**Authors:** Alessandra Bortoluzzi, Cecilia Beatrice Chighizola, Micaela Fredi, Elena Raschi, Caterina Bodio, Daniela Privitera, Arianna Gonelli, Ettore Silvagni, Marcello Govoni, Ilaria Cavazzana, Paolo Airò, Pier Luigi Meroni, Angela Tincani, Franco Franceschini, Silvia Piantoni, Fabio Casciano

**Affiliations:** ^1^ Rheumatology Unit, Department of Medical Sciences, University of Ferrara and Azienda Ospedaliero-Universitaria Sant’Anna, Cona, Italy; ^2^ Experimental Laboratory of Immunological and Rheumatologic Researches, Istituto Auxologico Italiano, IRCCS, Milan, Italy; ^3^ Rheumatology and Clinical Immunology Unit, Department of Clinical and Experimental Sciences, ASST Spedali Civili and University of Brescia, Brescia, Italy; ^4^ Department of Morphology, Surgery and Experimental Medicine and LTTA Centre, University of Ferrara, Ferrara, Italy

**Keywords:** angiogenic T cells, endothelial progenitor cells, immunosenescence, systemic lupus erythematosus, cardiovascular risk

## Abstract

Patients with systemic lupus erythematosus (SLE) have a significant increase in cardiovascular (CV) risk although they display a preserved number of circulating angiogenic CD3^+^CD31^+^CXCR4^+^ T cells (T_ang_), a subpopulation of T cells which promotes repair of damaged endothelium. This happens due to the concomitant expansion of a T_ang_ subset with immunosenescent features, such as the loss of CD28. Therefore, the aim of this study was to elucidate the interplay between T_ang_ subpopulations and endothelial cells in a group of young SLE patients without previous cardiovascular events. Twenty SLE female patients and 10 healthy controls (HCs) were recruited. Flow cytometric analysis of endothelial progenitor cells (EPCs) and T_ang_ subsets were performed and serum levels of interleukin (IL)-6, -8, matrix metalloproteinase (MMP)-9 and interferon (IFN)-*γ* were measured. Human umbilical vein endothelial cells (HUVECs) proliferation and pro-inflammatory phenotype in response to subjects’ serum stimulation were also evaluated. Results showed that the percentage of T_ang_ and EPC subsets was reduced in SLE patients compared with HCs, with a marked increase of senescent CD28^null^ cells among T_ang_ subset. SLE disease activity index-2000 (SLEDAI-2K) was inversed related to T_ang_ cells percentage. Furthermore, IL-8 serum levels were directly correlated with the percentage of T_ang_ and inversely related to the CD28^null^ T_ang_ subsets. We indirectly evaluated the role of the T_ang_ subset on the endothelium upon stimulation with serum from subjects with a low percentage of T_ang_ CD3^+^ cells in HUVECs. HUVECs displayed pro-inflammatory phenotype with up-regulation of mRNA for IL-6, intercellular adhesion molecule (ICAM)-1 and endothelial leukocyte adhesion molecule (ELAM)-1. Cell proliferation rate was directly related to IL-8 serum levels and EPC percentage. In highly selected young SLE patients without previous CV events, we found that the deterioration of T_ang_ compartment is an early event in disease course, preceding the development of an overt cardiovascular disease and potentially mediated by SLE-specific mechanisms. The overcome of the CD28^null^ subset exerts detrimental role over the T_ang_ phenotype, where T_ang_ could exert an anti-inflammatory effect on endothelial cells and might orchestrate *via* IL-8 the function of EPCs, ultimately modulating endothelial proliferation rate.

## Introduction

Systemic lupus erythematosus (SLE) is a polymorphic systemic autoimmune disease, burdened by a significant cardiovascular (CV) risk (1–3). The overall prevalence of vascular events ranges between 10 and 30%, with a 50-fold higher risk of myocardial infarction among young lupus women compared to age-matched controls (4). Patients also display a raised mortality due to vascular disease, and thrombotic events are the strongest predictors of death at five years from diagnosis (5). The increased CV burden manifests early in disease course, being largely attributable to endothelial activation and accelerated atherosclerosis (6). Indeed, patients with SLE have two-fold higher number of atherosclerotic plaques in the femoral arteries; at 5-year follow-up, 32% of SLE patients develop carotid atherosclerosis compared with 4% of controls (7, 8). Vascular damage is likely multifactorial, resulting from a complex interplay between traditional CV risk-factors and SLE-driven inflammation. Framingham risk-factors do not adequately account for cardiovascular disease (CVD) in lupus. Several SLE-associated items have been shown to contribute to the increased CV hazard such as disease activity and duration, renal involvement and steroid treatment (4). The presence of anti-phospholipid antibodies (aPL) represents an additional CV risk-factor in patients with SLE, as aPL not only trigger thrombotic events but also exert a direct role in the atherosclerotic process *via* the induction of endothelial activation (9). Given such important vascular morbidity and mortality, it is essential to investigate the mechanisms responsible for the increased CV burden in SLE.

Angiogenic T (T_ang_) cells are a subset of T cells (CD3^+^CD31^+^CXCR4^+^) that promotes vasculogenesis by orchestrating the function of endothelial progenitor cells (EPCs), and their characterization represents a promising field of research in CV medicine. Through the secretion of pro-angiogenic factors such as vascular endothelial growth factor (VEGF), interleukin (IL)-8 and matrix metalloproteinase (MMP)-9, T_ang_ cells exert a critical role in the formation of EPCs colonies, the differentiation of early EPCs and the potentiation of the function of early EPCs (10).

The pro-angiogenic potential of T_ang_ cells has been confirmed in *in vivo* models and in clinical studies conducted in the general population: the levels of T_ang_ cells are inversely related with age and CV risk-factors and correlate with EPC colony numbers, playing a role as predictive factor of CV events when reduced (10). Scant data are available in SLE where a conserved number of T_ang_ cells compared to healthy controls (HCs) have been found (11). An explanation to such apparent paradox comes from the observation that in SLE patients there is a significant expansion of a subpopulation within T_ang_ subset which displays immunosenescent characteristics with the loss of the co-stimulatory molecule CD28, required for T cell activation, survival and proliferation. Differently from the CD28^+^ counterpart, which likely represents the subgroup of protective T_ang_ cells, CD28^null^ T_ang_ cells exert detrimental effects on the endothelium (11). In fact, they display a cytotoxic profile, documented by the expression of perforin, granzyme B, CD56, and the secretion of significant amount of interferon (IFN)-*γ* (11), as previously demonstrated for CD4^+^CD28^null^ T cells (12).

Therefore, the aim of the IMMENSE (Interplay between iMMune and ENdothelial cells in mediating cardiovascular risk in Systemic lupus Erythematosus) study was to characterize T_ang_ subpopulations, investigating the crosstalk of T_ang_ with endothelial cells in young lupus patients without previous CV events.

## Materials and Methods

### Patients and Controls

From November 2017 to January 2019, a total of 20 patients aged less than 40 years and with a diagnosis of SLE according to the 1997 American College of Rheumatology (ACR) or the 2012 classification criteria for SLE (13, 14), attending the Rheumatology Unit of two tertiary referral centers for SLE, were recruited.

Exclusion criteria were any history of CVD including coronary heart disease (*i.e.* myocardial infarction, angina, coronary revascularization), cerebrovascular disease (*i.e.* stroke, transient ischemic attack), peripheral arterial disease, diabetes and chronic kidney disease (creatinine clearance <60 ml/min). Patients were matched for sex and age with 10 healthy controls (HCs) with no history of manifestations suggestive for systemic autoimmune disease and negative autoantibody profile. The study was approved by the Ethics Committee of each participating center (approval numbers 170187 [University of Ferrara], 2793 [University of Brescia] and 2017_10_24_3 [Istituto Auxologico Italiano]), and all patients provided written informed consent. The study was conducted in accordance with the Declaration of Helsinki.

Demographic features, including age, gender and ethnicity, were recorded. Data on the following CV risk-factors were collected: arterial hypertension (systolic blood pressure >140 mmHg and/or diastolic blood pressure >90 mmHg), dyslipidemia (blood total cholesterol, HDL-cholesterol and/or triglycerides outside normal limits), smoking (current or past), and obesity (body mass index >30). In order to investigate the presence of subclinical atherosclerosis, the carotid intima–media thickness (cIMT) was assessed by carotid ultrasound examination in the common carotid artery and the detection of focal plaques in the extracranial carotid tree. A commercially available scanner, (Mylab 70 Esaote, Genoa, Italy), equipped with 7–12 MHz linear transducer and the automated software guided technique radiofrequency “Quality Intima Media Thickness in real-time” (QIMT, Esaote, Maastricht, Netherlands) was used as elsewhere described in patients and controls (15). A cIMT greater than 0.9 mm was considered abnormal, and the presence of a plaque was identified by an IMT equal or greater than 1.5 mm, or by a focal increase in thickness of 0.5 mm or 50% of the surrounding cIMT value (16). Anti-hypertensive and lipid-lowering medications, anti-platelet or anti-coagulant agents were recorded in all patients and controls. Ongoing treatment with antimalarials, disease-modifying antirheumatic drugs or a combination of these agents was recorded together with the daily and the cumulative prednisone equivalent dose. In patients with SLE, the disease activity index-2000 (SLEDAI-2K) and the Systemic Lupus International Collaborating Clinics (SLICC) damage index (SDI) were calculated (17, 18).

### Blood Samples

Peripheral venous blood samples, from each patient and control, were collected into BD Vacutainer 6 ml tube containing EDTA for flow cytometry analysis, 3 ml tube containing sodium citrate for lupus anti-coagulant (LA) testing, 3 ml of serum-separating tube for serological assays and *in vitro* experiments (all from BD Biosciences, Franklin Lakes, NJ, USA). Aliquots of serum samples were stored at −20°C until assaying.

### Autoantibody Profile and Complement Dosage

All patients and controls were investigated for serum autoantibodies. Anti-nuclear antibodies (ANA) were tested in serum samples (5 μl) at indirect immunofluorescence on HEp-2 cells using the NOVA LiteTM ANA HEp-2 kit (Inova Diagnostics, San Diego, CA, USA) (positivity was defined at a titer ≥1:160) by manual reading with an epifluorescence microscope (Nikon Eclipse E400, Tokyo, Japan). Anti-dsDNA antibodies were detected by indirect immunofluorescence using Kallestad^®^ Crithidia luciliae (Bio-Rad Laboratories, CA, USA) with a cut-off titer of 1:10. Antibodies anti-extractable nuclear antigen (ENA) were detected with ANA Screen 9 Kit (Euroimmun AG, Lübeck, Germany) by ELISA using 1420 Multilabel Counter Victor3TM (PerkinElmer, UK) (positivity was defined at a titer >10 U/ml). The presence of LA was performed according to international guidelines (19). Anti-cardiolipin (aCL) antibodies, antibodies against beta2 glycoprotein I (anti-*β*2GPI IgG/IgM/IgA) and against *β*2GPI domain 1 (anti-D1 IgG) were detected in serum (30 μl/test) by a chemiluminescent immunoassay exploiting the BIO-FLASH technology using QUANTA Flash assays (Inova Diagnostics) (20). The cut-off values for aCL and anti-*β*2GPI IgG/IgM/IgA and anti-D1 IgG positivity were set at 20 chemiluminescent units (CUs), as recommended by the manufacturer.

C3 and C4 were measured by nephelometry; hypo-complementemia was defined by local laboratory reference values (C3 < 90 and C4 < 11 mg/dl detected in at least two separate occasions). CRP and ESR were considered as increased when above the cut-off defined by local routine laboratory.

### IL-6, MMP-9, IL-8, and IFN-*γ* Serum Levels

Serum levels of IL-6, IL-8, MMP-9, and IFN-*γ* were measured using commercially available ELISA kits (R&D Systems, Minneapolis, MN, USA) following the manufacturer’s instructions. The optical density (OD) values were evaluated at 450 nm using 1420 Multilabel Counter Victor3TM (PerkinElmer).

### Flow Cytometric Analysis

**Figure 1:**
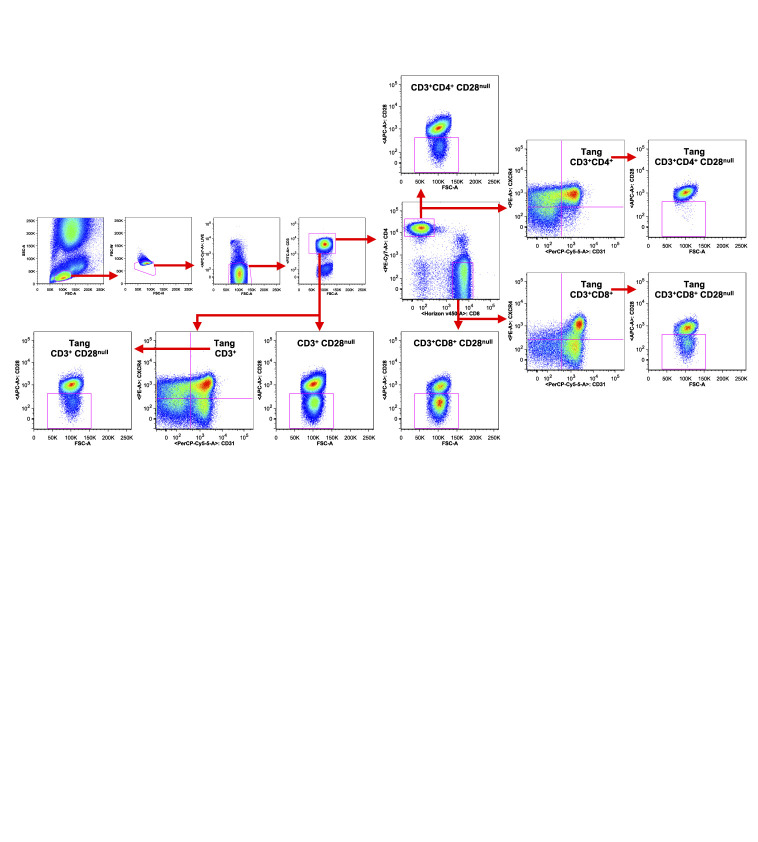
Gating strategy used to characterize T cell subpopulations. WBCs isolated from one representative SLE patient were analyzed by flow cytometry. Representative gating strategy analysis is shown. The axis scales for fluorescence are reported as log; the axis scales for SSC, FSC are reported as linear.

Peripheral venous blood samples were collected as above described and White Blood Cells (WBCs) were isolated for the analysis of flow cytometry using homemade red blood cells lysis buffer. Briefly, 1:1 part of blood:PBS (5 ml of blood; 5 ml of PBS) was added to nine parts (90 ml) of the homemade NH_4_Cl lysis solution (155 mM NH_4_Cl, 9.98 mM TrisBase and pH 7.4) and incubated at 37°C for 10 min. After the lysis step, samples were spinned down at 560 g for 5 min and further washed in PBS at 560 g for 5 min. The isolated WBCs were resuspended in PBS at the concentration of 1 × 10^6^ cells/ml. Next, the cells were stained as described below. Flow cytometric immunophenotyping was performed on WBC samples according to standard protocols with combinations of pre-titered fluorochrome-conjugated antibodies with FcR Blocking Reagent (Miltenyi Biotech GmbH, Bergisch Gladbach, Germany) at 4°C for 10 min as previously described (21, 22): CXCR4 PE (REA649), CD45 FITC (REA747), CD31 PerCP-Vio700 (REA730), CD3 FITC (REA613), CD4 PE-Vio770 (REA623), CD8 VioGreen (REA734), CD133 APC (AC133), CD34 PE (AC136), VEGFR2 PE-Vio770 (REA1116) (all from Miltenyi Biotech), and CD28 APC (28.2, BD Biosciences, Franklin Lakes, NJ, USA). In order to exclude dead cells, fixable viability dye eFluor™ 780 (eBioscience, San Deiego, CA, USA) was added to the staining mix. T_ang_ cells were defined by the expression of CD31 and CXCR4 among CD3^+^, CD3^+^CD4^+^ and CD3^+^CD8^+^ cells. The analysis of the expression of CD28 was done among different subpopulations (**Figure 1**). EPCs were defined in the mononuclear cellular gate as CD45dimCD34^+^ cells co-expressing CD133 (early EPCs CD133^+^) or CD133/VEGFR2 (late EPCs CD133^+^VEGFR2^+^) as elsewhere described (10, 23–27). The panels with antibody and fluorochromes used for each staining are listed in **Supplemental Information**. In each T_ang_ acquisition at least 1.5 × 10^5^ of lympho gate events were recorded and for EPC analysis at least 1 × 10^6^ lympho-mono gate events were acquired. Quality control included regular check-up with BD™ Cytometer Setup & Tracking Beads (BD Biosciences). To automatically assess fluorescence compensation, MACS Comp Bead Kits (Miltenyi Biotec) as well as the antibodies used in the assay were utilized. In order to evaluate T_ang_ and very rare cells (EPCs) in peripheral blood we used Fluorescence Minus One (FMO) control procedure to evaluate non-specific fluorescence when defining positive events as previously described (28), since it does not contain the antibody in the detector of interest representing the best control for any given marker of interest in a multicolor staining combination. Representative FMO for the analysis of very rare EPC subpopulation is shown in **Supplemental Figure 1**. All data collection was performed on FACS ARIAII using BD FACS Diva software (all from BD Biosciences), and data analysis was performed using the FlowJo software 9.9.6 (Tree Star, Ashland, OR, USA).

### Human Umbilical Vein Endothelial Cultures

Human umbilical vein endothelial cells (HUVECs) were isolated from normal term umbilical cord vein by type II collagenase perfusion (Worthington, Lakewood, NJ, USA). HUVEC cultures were maintained in complete E-199 medium (ThermoFisher Scientific, Waltham, MA, USA) supplemented with 20% heat inactivated FBS (PAA Laboratories-GE Healthcare, Toronto, Canada), 1% L-glutamine, 100 U/ml penicillin–streptomycin and 250 ng/ml Amphotericin B (all from MP Biomedicals, Santa Ana, CA, USA) at 37°C in CO_2_ 5%. Confluent cells were passaged with a 0.25% trypsin/EDTA (Gibco-ThermoFisher Scientific). HUVEC monolayers were incubated with sera from aPL-negative subjects at 1:2 dilution. In particular, subjects were stratified as follows: those with high percentage (>66th percentile) and the others with low percentage (<33th percentile) of T_ang_ CD3^+^ cells.

### IL-6, MMP-9, IFN-*γ*, ICAM-1, and ELAM-1 mRNA Expression Levels in HUVECs

The expression levels of IL-6, MMP-9, IFN-*γ*, inter-cellular adhesion molecule 1 (ICAM-1) and endothelial cell leukocyte adhesion molecule 1 (ELAM-1, alias E-Selectin encoded by the SELE gene) on HUVECs were evaluated by Real-Time PCR (RT-PCR) with Applied Biosystems 7500 Real-Time PCR System (ThermoFisher Scientific). HUVECs were resuspended in E-199 medium (ThermoFisher Scientific) containing 1% FBS added with 50% of serum from subjects and seeded in a 24-well plate at 5 × 10^3^ cells/well (300 μl). Internal controls at final concentration of 50 U/ml of recombinant human IL-1β, 10 ng/ml of recombinant human TNF-α (all from R&D System) and 1 μg/ml of LPS (Sigma-Aldrich, St. Louis, MO, USA) were used as positive controls, while E-199 medium containing 1% FBS provided the negative control. After 24 h of incubation at 37°C in CO_2_ 5%, HUVECs were harvested and total RNA was purified using Trizol Reagent (ThermoFisher Scientific). Amplification Grade DNase I (ThermoFisher Scientific) was used to eliminate residual genomic DNA. A reverse transcription reaction was performed using SuperScript™ First-Strand Synthesis System for RT-PCR (ThermoFisher Scientific). The PCR conditions were the following: 94°C for 10 min, followed by 45 cycles of 95°C for 15 s, 60°C for 60 s and 72°C for 30 s. Quantitative RT-PCR was performed on 100 ng of cDNA using TaqMan™ Universal PCR Master Mix, no AmpErase™ UNG (ThermoFisher Scientific) by ABIPRISM 7900 HT Sequence Detection System (ThermoFisher Scientific). Quantification of mRNA expression was performed with TaqMan^®^ Gene Expression Assay (ThermoFisher Scientific) for each target gene. Expression levels of target genes (*IL6*, *MMP9*, *IFNG*, *ICAM1* and *SELE*) were determined by the comparative Ct method normalizing the target to the endogenous gene (*GAPDH*). The following TaqMan^®^ Gene Expression assays were used: Hs00174131_m1 (*IL6*); Hs00957562_m1 (*MMP9*); Hs00989291_m1 (*IFNG*); Hs00164932_m1 (*ICAM1*); Hs00174057_m1 (*SELE*) and Hs99999905_m1 (*GAPDH*). Relative values of target to reference ratio were expressed as fold change (RQ).

### IL-6, MMP-9, IL-8, and IFN-*γ* Protein Expression Levels in HUVECs

The expression levels of IL-6, MMP-9, IL-8 and IFN-*γ* on HUVECs were evaluated by Western Blotting. HUVECs were resuspended in E-199 medium containing 1% FBS added with 50% of serum from subjects and seeded in a 24-well plate at 5 × 10^5^ cells/well (300 μl). Internal controls at final concentration of 50 U/ml of recombinant human IL-1β, 10 ng/ml of recombinant human TNF-α (all from R&D System) and 1 μg/ml of LPS (Sigma-Aldrich) were used as positive controls, while E-199 medium containing 1% FBS provided the negative control. After 24 h of incubation at 37°C in CO_2_ 5%, HUVECs were harvested and lysed using RIPA lysis buffer added with Protease and Phosphatase inhibitor cocktail (Sigma-Aldrich). Protein concentration was evaluated using BCA Protein Assay Kit (ThermoFisher Scientific). Equal amounts of proteins (10 μg/lane) were migrated in NuPAGE BIS-TRIS by 4–12% SDS-polyacrylamide pre-cast gel electrophoresis in MOPS buffer 1× for 50 min at 200 V and transferred to nitrocellulose for 7 min using iBlot Transfer Stacks Nitrocellulose and iBlot^®^ Gel Transfer Device (ThermoFisher Scientific). Membranes were blocked for 1 h at room temperature in PBS/0.1% Tween 20 (P/T) (Bio-Rad Laboratories) containing 5% non-fat milk powder (Mellin, Milan, Italy), and incubated overnight at 4°C with 1:1,000 of anti-human IL-6 (D3K2N), anti-human MMP-9 (D6O3H), anti-human IFN-*γ* (3F1E3) (all from Cell Signaling Technology, Danvers, MA, USA), anti-human IL-8 (6217, R&D Systems) and 1:2,000 of anti-human *α*-tubulin (B-5-1-2, Sigma-Aldrich). After washes, membranes were incubated in PT/5% non-fat milk powder plus anti-mouse or anti-rabbit Ig-G HRP-conjugated secondary antibodies (MP Biomedicals, Santa Ana, CA, USA) for 1 h at RT and revealed using ECL Plus Detection System (ThermoFisher Scientific). Signals were detected using radiographic films (Kodak, Rochester, NY, USA). Image J software (LI-COR Biosciences, Lincoln, NE, USA) was used to analyze and quantify densitometry values. Protein expression levels were normalized to the housekeeping gene, *α*-tubulin, and expressed as relative protein levels.

### HUVECs Proliferation Assay

The effect of stimulation with sera on HUVEC proliferation was determined using 3-(4,5-dimethylthiazol-2-yl)-2,5-diphenyl-2H-tetrazolium bromide (MTT) assay (Sigma-Aldrich) (29–31). HUVECs were resuspended in E-199 complete medium and incubated in a 96-well plate, 5 × 10^3^ cells/well for 24 h at 37°C in CO_2_ 5%. Then, the complete medium was removed, and the cells were cultured in E-199 medium with 1% FBS overnight at 37°C in CO_2_ 5%. Afterwards, the medium was removed, and the cells were stimulated with 100 μl of E-199 medium containing 1% FBS added with 10% of serum from subjects. Each treatment was performed in triplicate. After 24 and 72 h of incubation at 37°C in CO_2_ 5%, 10 μl of 10 mg/ml of MTT (final concentration 0.5 mg/ml) was added to each well and incubated at 37°C in CO_2_ 5% for 4 h. The formed formazan crystals were dissolved in dimethyl sulfoxide (150 μl/well) (Sigma-Aldrich) and the absorbance was read at 570 nm using a microplate scanning spectrophotometer (ELISA reader, OrganonTeknika, Netherlands). The percentage of proliferating cells was evaluated as follows: 100 × (absorbance of considered sample)/(absorbance of control). The experiments were performed on sub-confluent cell cultures in order to prevent contact inhibition, which can condition the results.

### Statistical Analysis

Statistics were calculated with GraphPad Prism 6 software. All data in box plot are presented as Tukey’s box plot where the higher whisker represent 75^th^ percentile plus 1.5 times interquartile range (IQR) and the lower whiskers represent 25^th^ percentile minus 1.5 times IQR. The Shapiro–Wilk test was used to evaluate the Gaussian distribution of overall data. Statistical comparisons between the different groups of subjects were calculated with non-parametric analyses (Mann–Whitney non-parametric U-test or Kruskal–Wallis test, when appropriated) when no Gaussian distribution was found and exact p values were obtained, otherwise T-students’ test was used. Correlation among variables was evaluated using the Spearman’s rank correlation coefficient or Pearson’s correlation coefficient according to the data’s Gaussian distribution. p values <0.05 were considered significant.

## Results

### Clinical Characteristics

Demographic data, clinical characteristics and pharmacological treatments of the study subjects are reported in **Table 1**. The autoantibody profiles of SLE patients and HCs are detailed in **Table 2**. The rate of positivity in non-criteria aPL test was very low and positivity for non-criteria aPL was never isolated.

**Table 1:** Demographic, clinical characteristics, and pharmacological treatments of the study subjects.

	SLE	HCs	p
Number of subjects	20	10	
F, n (%)	20 (100)	10 (100)	1.00
Age, mean (SD)	33 (5)	29.8 (3.8)	0.08
Caucasian, n (%)	17 (85)	10 (100)	0.53
CV risks factors			
BMI, mean (SD)	25.1 (4.5)	22.3 (1.7)	0.07
Obesity (BMI > 30), n (%)	3 (15)	0 (0)	0.53
Smoking (ongoing), n (%)	6 (30)	3 (30)	1.00
Smoking (past), n (%)	4 (20)	0 (0)	0.27
Hypertension, n (%)	1 (5)	0 (0)	1.00
Dyslipidemia, n (%)	2 (10)	0 (0)	0.54
Subclinical atherosclerosis (IMT > 0.9) or plaques, n (%)	0 (0)	0 (0)	1.00
Clinical and serological characteristics			
Disease duration, months (SD)	109 (56)	–	
SLEDAI-2K, mean (SD)	3.4 (2.6)	–	
SLEDAI-2K, range	0–10	–	
SLICC-SDI, mean (SD)	0.3 (0.4)	–	
Cutaneous involvement, n (%)	14 (70)	–	
Mucosal involvement, n (%)	5 (25)	–	
Articular involvement, n (%)	15 (75)	–	
Serositic involvement, n (%)	7 (35)	–	
Renal involvement, n (%)	6 (30)	–	
Neurological involvement, n (%)	2 (10)	–	
Hematological involvement, n (%)	13 (65)	–	
C3 mg/dl, mean (SD)	87.8 (21.8)	–	
C4 mg/dl, mean (SD)	16.6 (10)	–	
CRP mg/dl, mean (SD)	0.56 (1.04)	–	
ESR mm, mean (SD)	11 (0.3)	–	
Ongoing treatment			
Low dose aspirin, n (%)	10 (50)	–	
Oral anti-coagulant, n (%)	2 (10)	–	
Lipid-lowering drugs, n (%)	1 (5)	–	
Anti-hypertensive drugs, n (%)*	3 (15)	–	
Anti-malarial drugs, n (%)	19 (95)	–	
Steroids, n (%)	16 (80)	–	
Steroids, daily dosage (mg), mean (SD)	5 (4.2)	–	
Steroids, cumulative dosage (g), mean (SD)	15.6 (12.2)	–	
Disease modifying antirheumatic drugs, n (%)§	15/20 (75)	–	

*angiotensin-converting enzyme inhibitors in three patients for renal involvement, combined with one beta-blockers in one case; § azathioprine, cyclosporin, methotrexate, cyclophosphamide, mycophenolate or belimumab. p values < 0.05 were considered significant.

BMI, body max index; CRP, C-reactive protein; CV, cardiovascular; ESR, erythrocyte sedimentation rate; HCs, healthy controls; IMT, Intima Media Thickness; SD, standard deviation; SLEDAI-2K, SLE disease activity index-2000; SLICC-SDI, Systemic Lupus International Collaborating Clinics damage index; SLE, systemic lupus erythematosus.

**Table 2:** Autoantibody profiles of patients and controls.

	SLE	HCs
ANA, n (%)	20 (100)	1 (10)
Anti-extractable nuclear antigen positivity, n (%)	9 (45)	0 (0)
Anti-double stranded DNA, n (%)	13 (65)	0 (0)
LA, n (%)	8 (40)	0 (0)
aCL IgG, n (%)	7 (35)	1 (10)
aCL IgM, n (%)	3 (15)	0 (0)
aCL IgA, n (%)	2 (10)	0 (0)
anti-*β*2GPI IgG, n (%)	6 (30)	0 (0)
anti-*β*2GPI IgM, n (%)	1 (5)	0 (0)
anti-*β*2GPI IgA, n (%)	1 (5)	0 (0)
anti-*β*2GPI D1, n (%)	5 (25)	0 (0)

aCL, anti-cardiolipin antibodies; anti-β2GPI, anti-β2 glycoprotein I antibodies; D1, domain 1; HCs, healthy controls, ANA, anti-nuclear antibodies; LA, lupus anti-coagulant; SLE, systemic lupus erythematosus.

### Angiogenic T Cells and Peripheral Endothelial Progenitor Cells Are Decreased in SLE Patients

**Figure 2:**
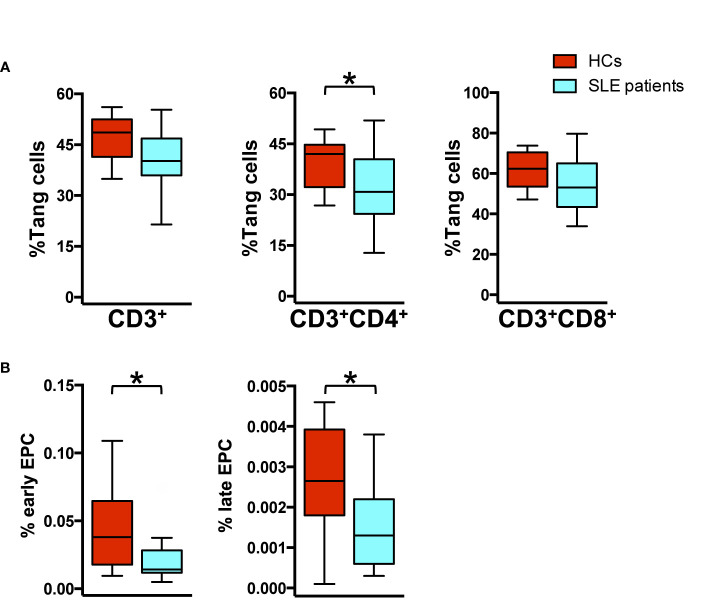
Impaired percentage of endothelial progenitor and T_ang_ cells in SLE patients. WBCs isolated from HC and SLE patients were analyzed by flow cytometry for the identification of T cell lineage, T_ang_ and EPC subpopulation. **(A)** The percentage of T_ang_ cells for each T cell subpopulation is represented as Tukey’s box plot. SLE patients show reduced percentage of T_ang_ cell subpopulations than HCs. **(B)** Differences in the early CD133^+^ and late CD133^+^VEGFR2^+^ EPC percentage among WBCs from HCs and SLE patients are represented as Tukey’s box plot. SLE patients show reduced percentage of EPC subpopulation than HCs. The y axis scale is reported as linear. Statistical analysis of the differences was performed by Mann–Whitney test. p values <0.05 were considered significant: *p < 0.05.

First, we characterized WBCs for the expression of T_ang_ and EPC subpopulations in our cohort. Phenotypic characterization of WBCs showed that the percentage of T_ang_ CD3^+^CD4^+^ subpopulation was reduced in SLE patients as compared to HCs (p = 0.04). Similar results were observed for CD3^+^ (p = 0.07) and CD3^+^CD8^+^ (p = 0.12) T_ang_ cells (**Figure 2A**). SLE patients showed a significantly lower percentage of EPCs CD133^+^ (p = 0.027) and particularly of EPCs CD133^+^VEGFR2^+^ (p = 0.012) when compared to HCs (**Figure 2B**) (**Supplemental Table 1**).

Since aPL antibodies are additional CV risk-factors, we investigated the percentage of T_ang_ and EPCs among SLE patients positive for aPL; however, our analysis showed that there was no significant difference in the percentage of circulating T_ang_ and EPC subpopulations (data not shown).

Furthermore, correlations between the percentage of T_ang_ and EPC subpopulations were not found among patients or HCs.

### Angiogenic CD28^null^ T Cells Are Increased in SLE Patients

**Figure 3:**
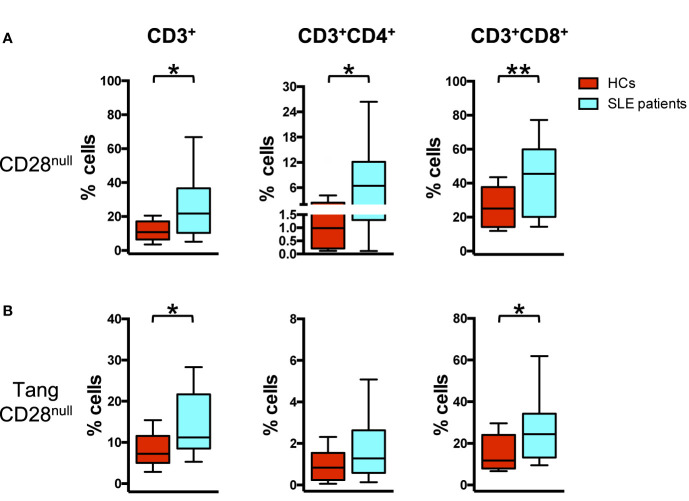
Senescent angiogenic T cells characterize SLE patients. WBCs isolated from HC and SLE patients were analyzed for the differences in the percentage of CD28^null^ cells in T_ang_ and parent T cell subpopulations using flow cytometry. **(A)** The percentages of CD28^null^ cells for each T cell subpopulation are represented as Tukey’s box plot. SLE patients show higher percentage of CD28_null_ cell subpopulations than HCs. **(B)** The percentages of CD28^null^ cells within each T_ang_ cell subpopulation are represented as Tukey’s box plot. SLE patients show higher percentage of CD28^null^ T_ang_ cell subpopulations than HCs. The y axis scale is reported as linear. Statistical analysis of the differences was performed by Mann–Whitney test. p values <0.05 were considered significant: *p < 0.05, **p < 0.01.

Analyzing the senescent profile of T cells in relation to the expression of CD28 receptor, an increased percentage of CD28^null^ cells was observed in SLE compared to HCs (p = 0.002) in CD3^+^ peripheral blood T cells. This increase was mainly evident in T CD3^+^CD8^+^ subpopulation (p = 0.006), although was also present in the T CD3^+^CD4^+^ subset (p = 0.020) (**Figure 3A**).

Moreover, the percentage of senescent CD28^null^ cells within the T_ang_ CD3^+^ subset was increased in SLE patients (p = 0.019) when compared to HCs. In particular, among T_ang_ CD3^+^ cells, the down regulation of CD28 expression was mainly evident among T_ang_ CD8^+^ cells (p = 0.04) (**Figure 3B**, **Supplemental Table 2**) and among T_ang_ CD4^+^CD8^+^ cells (p = 0.01) (**Supplemental Figure 2B**).

### Circulating Levels of Peripheral Angiogenic T Cells and Endothelial Progenitor Cells Decrease According to the Disease Activity

**Figure 4:**
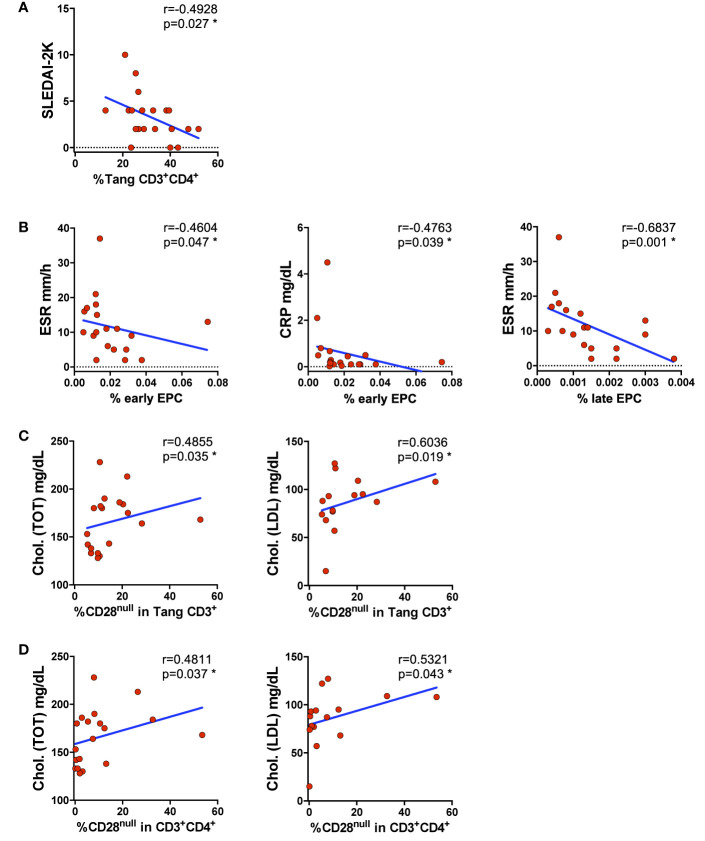
Impaired percentage of circulating EPCs and T_ang_ is disease activity related. WBCs isolated from SLE patients were analyzed for T_ang_ and senescent CD28^null^ T_ang,_; EPC subsets and the percentages of cells were correlated with clinical findings. **(A)** Correlation between the percentages of T_ang_ CD3^+^CD4^+^ cells and SLEDAI-2K is shown. The percentage of T_ang_ CD3^+^CD4^+^ cells inversely correlates with SLEDAI-2K. **(B)** Correlation between the percentages of EPCs depicted as early CD133^+^ or late CD133^+^VEGFR2^+^ cells and systemic inflammatory markers is shown. The percentage of EPCs inversely correlates with the serum levels of systemic inflammatory markers CRP and ESR. **(C)** Correlation between the percentages of CD28^null^ within T_ang_ CD3^+^ cells and total or LDL cholesterol is shown. The percentages of the senescentCD28^null^ T_ang_ cells directly correlate with cholesterol serum levels. **(D)** Correlation between the percentage of CD28^null^ cells within CD3^+^CD4^+^ subpopulation and total or LDL cholesterol is shown. The percentages of senescentCD28^null^ T_ang_ CD3^+^CD4^+^ cells directly correlate with cholesterol serum levels. The axis scales are reported as linear. Correlations are expressed as Spearman r values, p values <0.05 were considered significant: *p < 0.05.

Analyzing the variation of the percentage of T_ang_ and EPCs subsets in relation to clinical findings, we found that the percentages of T_ang_ CD3^+^CD4^+^ cells were inversely related to SLEDAI-2K (**Figure 4A**). The same could be seen for EPCs in relation to inflammatory markers, where the percentages of early EPCs CD133^+^ cells were inversely associated to ESR and CRP. We also observed a positive correlation comparing the percentage of late EPCs CD133^+^VEGFR2^+^ subset with ESR (**Figure 4B**). No correlations were found between T_ang_ percentage and ESR or CRP.

It is well established that the lipid profile influences the CV risk, but is unknown if it could be related with the CD28^null^ senescent status of T_ang_ cells in SLE patients. Analyzing the T_ang_ senescent status in relation to lipid profile, a direct correlation between the serum level of total cholesterol and percentage of CD28^null^ T_ang_ CD3^+^ and CD28^null^ T CD3^+^CD4^+^ cells was observed. Similarly, LDL cholesterol serum levels were positively correlated with the percentage of senescent CD28^null^ in T_ang_ CD3^+^ or CD4^+^ cells (**Figures 4C, D**) (**Supplemental Table 3**).

No correlations were found between cell percentage numbers and BMI values (data not shown).

### The Percentage of Circulating T_ang_ Cells Directly Correlate With Serum Levels of IL-8

**Figure 5:**
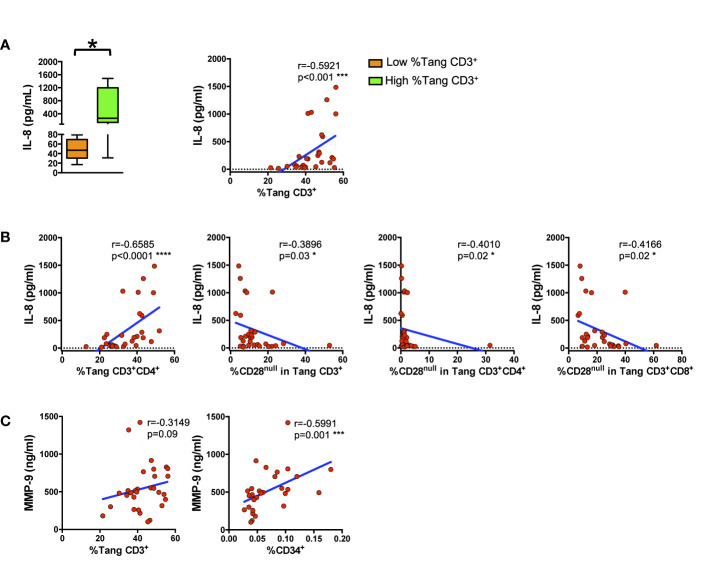
Circulating proangiogenic factors reflect angiogenic circulating cells related compartments. WBCs isolated from subjects were analyzed for T cell lineage. T_ang_ and EPC markers and the percentages of cells were correlated with IL-8 and MMP-9 serum levels. **(A)** The differences between the serum IL-8 levels of subjects with low percentage of circulating T_ang_ CD3^+^ cells and of subjects with high percentage of circulating T_ang_ CD3^+^ cells are represented as Tukey’s box plot (left panel). Right panel shows the correlation between the percentages of T_ang_ CD3^+^ cells and IL-8 serum level in the whole cohort. IL-8 serum levels directly correlate with the percentages of T_ang_ CD3^+^ cells. **(B)** The correlation between serum levels of IL-8 and the percentage of T_ang_ CD3^+^CD4^+^ and of CD28^null^ cells within each T_ang_ cell subpopulation in both HCs and SLE patients are shown. IL-8 serum levels inversely correlate with the percentage of senescent CD28^null^ T_ang_ cells. **(C)** The correlation between MMP-9 and the percentage of circulating CD34^+^ or T_ang_ CD3^+^ cells for the whole cohort are shown. MMP-9 serum levels directly correlate with the percentage of CD34^+^ or T_ang_ cells. The axis scales are reported as linear. Statistical analysis of the differences was performed by Mann–Whitney test. Correlations are expressed as Spearman r values, and significance levels are indicated. p values <0.05 were considered significant: *p < 0.05, ***p < 0.001, ****p < 0.0001.

IL-8 and MMP-9 modulate endothelial homeostasis. Therefore, we analyzed if the T_ang_ subpopulation and the senescent CD28^null^ subset could be associated with serum levels of these factors. As above detailed, SLE patients and HCs were stratified by the tertile of the percentage of circulating T_ang_ CD3^+^ cells (low, <33^th^ percentile; high, >66^th^ percentile). Individuals with high T_ang_ CD3^+^ cells percentage displayed higher levels of IL-8 (**Figure 5A** left panel). Accordingly, IL-8 serum levels significantly correlated with the percentage of circulating T_ang_ CD3^+^ cells in the whole cohort (**Figure 5A**, right panel). In addition, serum levels of IL-8 directly correlated with the percentage of T_ang_ CD3^+^CD4^+^ cells while, as expected, were overall inversely related to the CD28^null^ T_ang_ subpopulations (CD3^+^, CD3^+^CD4^+^, CD3^+^CD8^+^) (**Figure 5B**). SLE patients and HCs with high percentage of T_ang_ CD3^+^ cells presented serum levels of IL-6, MMP-9 and IFN-*γ* similar to those with lower percentage of T_ang_ CD3^+^ cells (data not shown). However, a trend towards statistical significance emerged when MMP-9 serum levels were correlated to T_ang_ CD3^+^ cells (**Figure 5C**, left panel). Furthermore, MMP-9 levels were directly related to the percentage of the pluripotent bone marrow progenitor CD34^+^ (**Figure 5C**, right panel) (**Supplemental Table 4**). Details of T_ang_ subsets in SLE patients and HCs stratified by the tertile of the percentage of circulating T_ang_ CD3^+^ cells are shown in **Supplemental Table 5**.

HCs displayed significantly higher serum levels of IL-6, IL-8 and MMP-9 compared to SLE patients, whereas serum IFN-*γ* was similar between the two groups of subjects (**Supplemental Table 6**).

### A Pro-Inflammatory Phenotype in HUVECs Emerges Upon Stimulation With Sera From Subjects With Low Percentage of T_ang_ Cells

**Figure 6:**
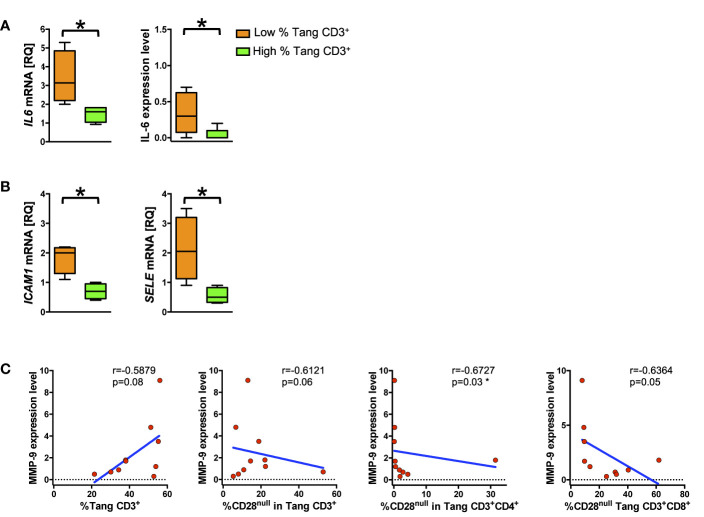
Low percentage of circulating T_ang_ cells correlates with pro-inflammatory endothelial phenotype. HUVECs were treated for 24 h with serum from subjects (SLE patients and controls) with high or low percentage of T_ang_ CD3^+^ and then analyzed for mRNA and protein expression level. **(A)** The differences between of mRNA and protein expression level of *IL6* from HUVECs treated with serum of subject with low percentage of circulating T_ang_ CD3^+^ cells and HUVECs treated with serum of subjects with high percentage of circulating T_ang_ CD3^+^ cells are shown. Data are represented as Tukey’s box plot. HUVECs stimulated with serum from subjects with low percentage of T_ang_ CD3^+^ cells show higher IL-6 expression. **(B)** The differences of ICAM1 (left panel) and *SELE* (right panel) mRNA expression between HUVECs treated with serum of subject with low percentage of circulating T_ang_ CD3^+^ cells and HUVECs treated with serum of subject with high percentage of circulating T_ang_ CD3^+^ cells are represented as Tukey’s box plot. HUVECs stimulated with serum from subjects with low percentage of T_ang_ CD3^+^ cells show higher adhesion molecules expression. **(C)** HUVEC_S_ were stimulated for 24 h with serum from subjects with high or low percentage of T_ang_ CD3^+^, and the MMP-9 expression levels were correlated with the percentage of circulating T_ang_ and CD28^null^ T_ang_ cells. The expression of MMP-9 is related with the percentage of T_ang_ cells and declines according to the percentage of circulating CD28^null^ T_ang_ cells. The axis scales are reported as linear. Relative Quantification (RQ) expresses fold of change of target to reference. Statistical analysis of the differences was performed by Mann–Whitney test. Correlations are expressed as Spearman r values, and significance levels are indicated. p values <0.05 were considered significant: *p < 0.05.

To explore the interplay of T_ang_ cells with the endothelium, we analyzed the response of HUVECs to the stimulation with serum from subjects (patients and HCs) stratified according to their percentage of circulating T_ang_ CD3^+^ cells as above described. HUVECs treated with sera from subjects with a low percentage of T_ang_ CD3^+^ cells displayed higher mRNA as well as protein expression levels of IL-6 than cells treated with sera from subjects with higher percentage of T_ang_ CD3^+^ cells (p = 0.02 and p = 0.04, respectively) (**Figure 6A**). Similarly, treatment with sera from subjects with a low percentage of T_ang_ CD3^+^ cells resulted in higher mRNA expression levels of both ICAM-1 and ELAM-1 compared to HUVECs treated with sera from subjects with higher percentage of T_ang_ CD3^+^ cells (**Figure 6B**). Although we did not detect differences in IL-8, MMP-9 and IFN-*γ* expression levels when HUVECs were stimulated with sera from subjects with low or high percentage of T_ang_ CD3^+^ cells, suggestive data emerged for MMP-9. Indeed, protein expression levels were positively related to the percentage of T_ang_ CD3^+^ cells approaching statistical significance and were inversely correlated to the percentage of CD28^null^ T_ang_ subpopulation (**Figure 6C**) (**Supplemental Table 7**).

Upon treatment with serum samples from SLE patients and HCs, HUVECs presented similar mRNA and protein expression levels of study mediators (**Supplemental Tables 8** and **9**).

### HUVECs Proliferation Positively Correlates With IL-8 Serum Levels and With Endothelial Progenitor Cells

**Figure 7:**
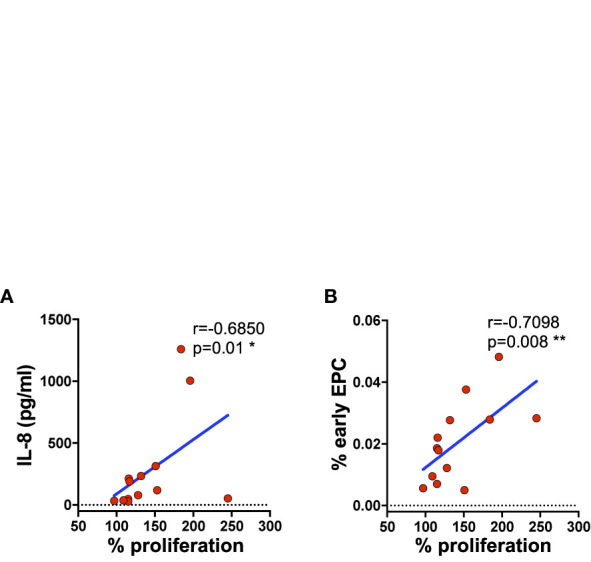
HUVEC cell proliferation directly correlates with the serum concentration of IL-8 and the percentage of circulating subpopulation. HUVEC proliferation was evaluated by MTT after 72 h of incubation with serum from subjects (SLE patients and controls) with high and low percentage of T_ang_ CD3^+^. Correlation between the percentage of proliferating cells and IL-8 serum levels **(A)** or the percentage of EPCs CD133^+^ subpopulation **(B)** is shown. Correlations are expressed as Spearman r values, and significance levels are indicated. p values <0.05 were considered significant: *p < 0.05, **p < 0.01.

To investigate further the protective role of T_ang_ over endothelial cells we analyzed the proliferation of HUVECs in response to serum stimulation by MTT assay. Although no differences in HUVEC proliferation upon stimulation with serum from subjects with low or high percentage of T_ang_ CD3^+^ cells were observed [median (IQR) 57% (34–82) and 67% (62–84), respectively] at 24 h, at 72 h relevant correlation were found. Indeed, as shown in **Figure 7**, endothelial cell proliferation correlated positively with IL-8 serum levels and early EPCs CD133^+^ subset at 72 h (**Supplemental Table 10**).

Treatment with sera from SLE patients and HCs resulted in a similar modulation of the proliferation rate of HUVECs (**Supplemental Table 11**).

## Discussion

The IMMENSE study provides novel insights into the evaluation of CV subclinical alterations in SLE by unveiling some potential aspects of the complex interplay between T_ang_, EPCs, and endothelial cells. The focus of the study was on circulating T_ang_ cells, which were recently demonstrated to be a potentially useful biomarker reflecting vascular alterations in CV and autoimmune diseases (10, 11, 32, 33).

In our highly selected young SLE patients without previous CV events and a low rate of traditional CV risk-factors, we found a mild decrease of T_ang_ cells in patients in comparison with HCs, confirming our previous observation (34). Notably, we detected for the first time that T_ang_ reduction in SLE was particularly evident among CD4^+^ subpopulation and inversely related to disease activity as evaluated by SLEDAI-2K. Conversely, the only two other available studies reported a similar percentage of T_ang_ cells between SLE patients and HCs (11, 35), pointing out a decrease of CD28^+^ T_ang_ cells in SLE patients with CVD. These results suggested that the CD28 expression should be used to redefine the pro-angiogenic T_ang_ cells (12). A reduced proportion of T_ang_ cells has been reported also in patients with rheumatoid arthritis (RA), especially in those who had experienced CV events (36).

Given the relevance of the immunosenescent phenotype of T_ang_ cells in relation to CV burden, we then focused on CD28^null^ T_ang_ subsets, reporting an increase in CD28^null^ T_ang_ among CD3^+^, CD4^+^ and CD8^+^ subpopulations in SLE patients. Our results agree with the studies by Lopez and coworkers, who were the first to describe an increase of this cell subpopulation among lupus subjects (11, 37). Noteworthy, they observed that the increase of CD28^null^ T_ang_ cells was most relevant among patients with CVD, independently from age, gender, disease duration, disease activity, comorbidities, and use of drugs. Interestingly, CD28^null^ T_ang_ cells appeared to be related to high circulating levels of pro-inflammatory mediators (TNF-α, IFN-α) and to the positivity for anti-dsDNA and anti-Ro autoantibodies. Overall these data confirmed the evidence raised in the general population on a close relation between CD28^null^ T cells percentage and CV events: circulating levels of CD4^+^CD28^null^ T cells were expanded in a cohort of patients with unstable angina, provided a strong and independent predictor of mortality in patients with heart failure and were found as tissue-infiltrating T cells in unstable atherosclerotic plaques (37–40). The evidence of the detrimental role of this cell subset on CV system has been confirmed even in autoimmune diseases other than SLE. Some authors found an increased number of CD4^+^CD28^null^ T cells as a possible distinctive feature of RA patients with high CV risk, measured by the cIMT and by the brachial artery flow mediated vasodilatation (41).

The data on the decrease of T_ang_ and the raise of CD28^null^ T_ang_ cells highlighted in the IMMENSE study are particularly relevant given the highly selected composition of our study group, which included exclusively young patients without previous CV events and a low rate of conventional CV risk-factors. Indeed, our observation suggested that the deterioration of the T_ang_ compartment is an early event in disease course, preceding the development of an overt CVD. The inverse correlation of T_ang_ cells with disease activity suggests that SLE-specific mechanisms could mediate the deterioration of this T cell subset. This should not be surprising, as immunosenescence might be driven not only by aging but also by repeated antigen stimulations as happens in systemic autoimmune diseases such as SLE (42). Besides the loss of CD28, several additional processes occur, influencing the number and function of circulating immune cells, such as telomere attrition and DNA damage (43).

We could not observe any significant correlation between CD4^+^CD28^null^ T cells and disease specific markers, including criteria or non-criteria aPL. However, CD28^null^ T_ang_ CD3^+^, as well as CD28^null^ T_ang_ CD3^+^CD4^+^ cells, were directly correlated with total and LDL cholesterol serum levels. Interestingly, this clinical correlation is hereby described for the first time in SLE patients, further reinforcing the importance of the T_ang_ loss of CD28 in relation to CV risk. In 2015, a negative correlation between total cholesterol serum levels and T_ang_ CD3^+^ cells had been found in a cohort of healthy subjects, but not confirmed among RA patients (36). The differences between healthy subjects and patients might be ascribed to the striking prevalence of dyslipidemia in RA subjects enrolled in that study, which was as high as 36% (36).

Moreover, our study shed light on the interplay between T_ang_ and endothelial cells. Hence, T_ang_ cell percentages were found to directly correlate with serum levels of IL-8 and MMP-9, two well-characterized pro-angiogenic mediators (44). As expected, the CD28^null^ counterpart of T_ang_ cells was negatively correlated with IL-8 serum levels, reinforcing the potential involvement of this inflammatory subset in mediating endothelial dysfunction (11, 36–39). *In vitro* experiments were conducted using sera from subjects stratified upon the percentage of CD3^+^ T_ang_ cells: conclusions on the effects of CD3^+^ T_ang_ cells on endothelial cell phenotype and proliferation could thus be derived only indirectly. The evidence that sera from subjects with a low percentage of T_ang_ cells had detrimental effects on HUVEC proliferation and phenotype, differently from sera of those with high T_ang_ cell number, is in agreement with the notion that T_ang_ cells exert a proangiogenic potential. Observed results might have been biased by additional cellular or soluble mediators: indeed, surely T_ang_ do not provide the only determinant of serum cytokine levels and several additional cell types contribute to circulating cytokines such as NK, monocytes and dendritic cells (45, 46).

In our study, we found a lower percentage of EPCs in SLE patients in comparison with HCs, in agreement with the majority of studies on lupus subjects, possibly due to increased apoptosis. Indeed, CD34^+^AnnexinV^+^ circulating cells were expanded in a cohort of SLE patients in clinical remission, compared with controls (47). Furthermore, one study reported also increased apoptosis of hematopoietic stem cells (HSCs) and decreased CD34^+^ HSCs in the bone marrow of patients with active SLE, which could affect the CD34^+^VEGFR2^+^ EPCs (48). Decreased percentage of EPCs was described in SLE patients without any apparent clinical correlation, supporting the hypothesis of chronically decreased levels throughout the disease (49). In our patients, all with low activity and damage indexes, no correlation was found between EPCs percentage and clinical features. Recent studies in SLE patients showed also an impairment in EPCs’ function with a decreased ability to produce VEGF, to migrate and to proliferate (50).

The limitations of the study consist in the low number of enrolled patients and, in particular, the low number of aPL positive patients which prevented further analysis. The low disease activity of enrolled patients might account for the unexpectedly lower levels of serum cytokines that we observed in SLE subjects compared to HCs and might possibly have impinged the accuracy of statistical analysis when evaluating the association between cell subsets and SLEDAI-2K.

However, the strength of the IMMENSE study is related to the enrollment of young female SLE patients and matched HCs, with a low rate of modifiable CV risk-factors, in order to overcome possible confounders in the analysis of CV parameters. Furthermore, enrolled patients had a clinically inactive–serologically active SLE being the best candidates to evaluate, since disease was under control with first-line therapies, minimizing the possible interference of therapies with the number of circulating cells.

## Conclusions

As a whole, the IMMENSE study supports the hypothesis that T_ang_ subpopulation potentially exerts a key role in mediating CV risk among SLE patients, confirming the heterogeneous nature of these cells (51). This work further unveils the complex interplay between T_ang_ and EPC subsets, even though we acknowledge that future studies exploiting more sophisticated experimental approaches are needed to gain mechanistic insights into the crosstalk between T_ang_ and endothelial cells.

Most importantly, we observed that the percentage of circulating pro-angiogenic T_ang_ decrements very early in disease course, with an increase in the rate of senescent inflammatory CD28^null^ subset. These modulations of T_ang_ cell percentage might account for detrimental effects on the endothelium. Our preliminary and descriptive data suggest that T_ang_ might exert their effects on the endothelium *via* the pro-angiogenic mediators IL-8 and MMP-9, as documented by the following lines of evidence: i) the strong correlation between T_ang_ and these factors, ii) the inverse correlation of IL-8 with CD28^null^ T_ang_ subpopulations, iii) the perturbed endothelial phenotype induced by the stimulation with sera from subjects with low number of circulating T_ang_ CD3+ cells, and iv) the correlation between endothelial cell proliferation and IL-8 serum levels.

Our observation confirms the relationship between T_ang_ subsets and the endothelium in SLE, highlighting the necessity to extend these observations longitudinally in wider cohorts of lupus patients, potentially leading to the identification of surrogate markers to early stratify SLE subjects according to the future risk of CV events.

## Data Availability Statement

All datasets presented in this study are included in the article/**Supplementary Material**.

## Ethics Statement

The studies involving human participants were reviewed and approved by the Ethics Committee of each participating center [approval numbers 170187 (University of Ferrara), 2793 (University of Brescia) and 2017_10_24_3 (Istituto Auxologico Italiano)]. The patients/participants provided their written informed consent to participate in this study.

## Author Contributions

AB, CC, and MF designed the study, selected the patients and control subjects to be recruited in the study, collected clinical data, acquired funding, and wrote the final version of the manuscript. CC collected *in vitro* data and performed the statistical analysis. ER performed experiments, analyzed the data, and contributed to the writing of the manuscript. DP, CB, and AG performed experiments and contributed to the writing of the manuscript. ES, MG, PA, AT, and FF participated in data interpretation and contributed to the writing of the manuscript. IC designed the study and contributed to the writing of the manuscript. PM contributed to the writing of the manuscript. FC and SP designed the study, performed experiments, collected flow cytometric data, coordinated the research activities, and wrote the manuscript. All authors contributed to the article and approved the submitted version.

## Funding

This work was supported by a grant from Fondazione Italiana per la Ricerca sull'Artrite (FIRA Onlus) and University of Ferrara (FAR 2020 N-FAR.L-BA_003).

## Conflict of Interest

The authors declare that the research was conducted in the absence of any commercial or financial relationships that could be construed as a potential conflict of interest.

## Abbreviations

aCL, anti-cardiolipin antibodies; ACR, American College of Rheumatology; aPL, anti-phospholipid antibodies; BMI, body mass index; cIMT, carotid intima-media thickness; CRP, C-reactive protein; CV, cardiovascular; CVD, cardiovascular disease; ELAM-1, endothelial cell leukocyte adhesion molecule 1; ENA, extractable nuclear antigen; EPCs, endothelial progenitor cells; ESR, erythrocyte sedimentation rate; FMO, fluorescence minus one; HCs, healthy controls; HDL, high-density lipoprotein; HSCs, hematopoietic stem cells; HUVECs, Human umbilical vein endothelial cells; ICAM-1, inter-cellular adhesion molecule 1; IFN-, interferon; IL-, interleukin; IMT, intima-media thickness; IQR, interquartile range; LA, lupus anti-coagulant; LDL, low-density lipoprotein; MMP, matrix metalloproteinase; MTT, 3-(4,5-dimethylthiazol-2-yl)-2,5-diphenyl-2H-tetrazolium bromide; RA, rheumatoid arthritis; SLE, systemic lupus erythematosus; SLEDAI-2K, systemic lupus erythematosus disease activity index 2000; T_ang_, angiogenic T cell; TNF-, tumor necrosis factor; VEGFR2, vascular endothelial growth factor receptor 2; WBCs, white blood cells.
