# Supplementary material for: The IMMENSE Study: The Interplay Between iMMune and ENdothelial Cells in Mediating Cardiovascular Risk in Systemic Lupus Erythematosus
Source: Front Immunol. 2020 Oct 29;11:572876. doi: 10.3389/fimmu.2020.572876 (PMC7658008; doi:10.3389/fimmu.2020.572876)
Supplement: Supplementary file 1 [file DataSheet_1.pdf]

The IMMENSE study: the Interplay between iMMune and ENdothelial cells in mediating cardiovascular risk in Systemic lupus Erythematosus.

Alessandra Bortoluzzi[1,§], Cecilia Beatrice Chighizola[2,§], Micaela Fredi[3,§], Elena Raschi[2], Caterina Bodio[2], Daniela Privitera[2], Arianna Gonelli[4], Ettore Silvagni[1], Marcello Govoni[1], Ilaria Cavazzana[3], Paolo Airò[3], Pier Luigi Meroni[2], Angela Tincani[3], Franco Franceschini[3], Silvia Piantoni[3,*,£] and Fabio Casciano[4,*].

1 Rheumatology Unit, Department of Medical Sciences, University of Ferrara and Azienda Ospedaliero-Universitaria Sant'Anna, Cona, Italy

2 Experimental Laboratory of Immunological and Rheumatologic Researches, Istituto Auxologico Italiano, IRCCS, Milan, Italy.

3 Rheumatology and Clinical Immunology Unit, Department of Clinical and Experimental Sciences, ASST Spedali Civili and University of Brescia, Brescia, Italy.

4 Department of Morphology, Surgery and Experimental Medicine and LTTA Centre, University of Ferrara, Ferrara, Italy.

§ These authors have contributed equally to this work.

* These authors shared senior authorship

£ Corresponding author:

Silvia Piantoni [e-mail: slv.piantoni@gmail.com]. Rheumatology and Clinical Immunology Unit, Spedali Civili and University of Brescia, Piazzale Spedali Civili 1, 25123 Brescia, Italy.

Supplementary Information

Tang:
CD3 FITC [REA613]
CXCR4 PE [REA649]
CD31 PerCP-Vio700 [REA730]
CD4 PE-Vio770 [REA623]
CD28 APC [28.2]
LIVE/DEAD eFluor 780
CD8 VioGreen [REA734]

EPC:
CD45 FITC [REA747]
CD34 PE [AC136]
VEGFR2 PE-Vio770 [REA1116]
CD133 APC [AC133]
LIVE/DEAD eFluor 780

Supplemental material 1. Panels with antibody and fluorochromes used for the flow cytometric analysis.

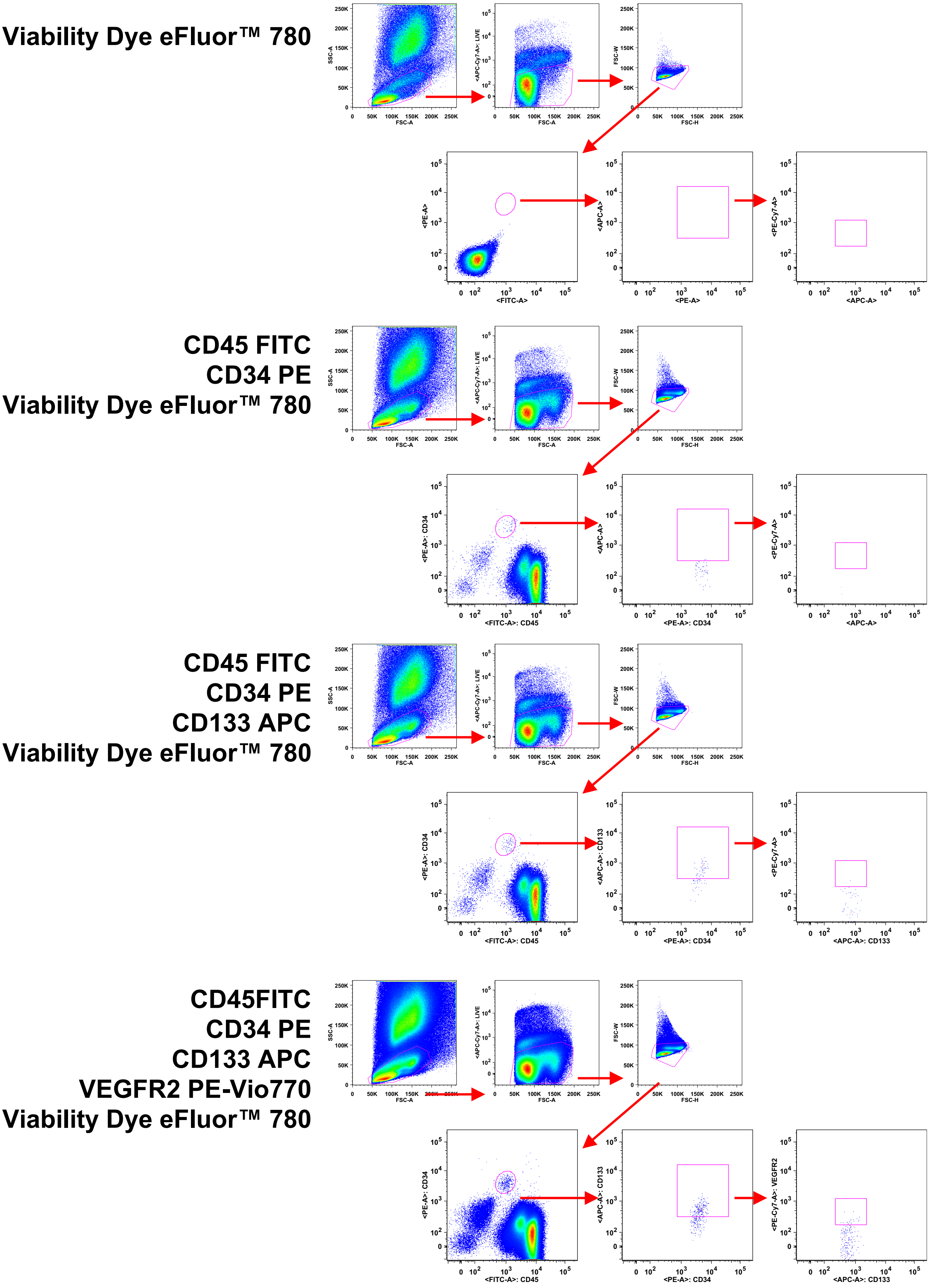

Supplemental figure 1. Example of Fluorescence Minus One [FMO] staining.
WBCs isolated from one representative healthy donor were analyzed for the expression of EPCs by flow cytometry.
The axis scales for fluorescence are reported as log, the axis scales for SSC, FSC are reported as linear.

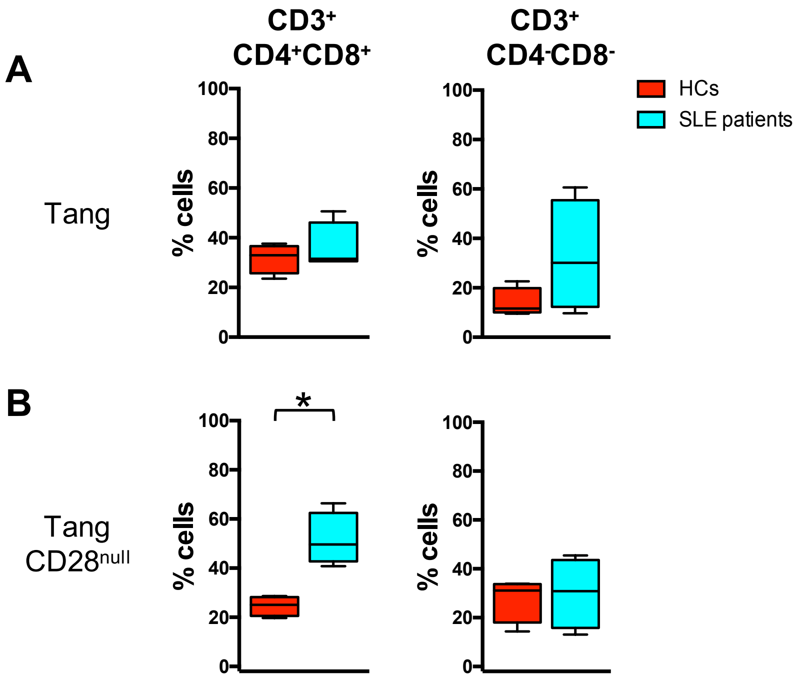

Supplemental figure 2. Analysis of double positive [CD4$^+$CD8$^+$] and double negative [CD4$^-$ CD8$^-$] Tang subpopulations.

WBCs isolated from HCs and SLE patients were analyzed for the differences in the percentage of Tang cells and CD28null cells in Tang subpopulations using flow cytometry.

(A) The percentage of T_{ang} cells for each T cell subpopulation is represented as Tukey's boxplot.

(B) The percentages of CD28^{null} cells within each T_{ang} cell subpopulation is represented as Tukey's boxplot. SLE patients show higher percentage of double positive CD4$^+$CD8$^+$ CD28^{null} T_{ang} cell subpopulations than HCs.

The axis scales are reported as linear. Statistical analysis of the differences was performed by Mann-Whitney test. p values <0.05 were considered significant: * p<0.05

	% Tang $CD3^+$	% Tang $CD3^+CD4^+$	% Tang $CD3^+CD8^+$	% early EPC	% late EPC
**HCs**	48.6 [41.4 - 52.4]<br>47.1±7.1	42.0 [26.8 - 44.7]<br>39.6±7.4	62.3 [47.2 - 70.5]<br>61.6±9.1	0.038 [0.009 - 0.064]<br>0.044±0.030	0.002 [0.001 - 0.003]<br>0.002±0.001
**SLE**	40.2 [35.9 - 46.8]<br>41.0±9.2	35.8 [24.3 - 40.4]<br>32.7±10.3	53.0 [33.9 - 65.0]<br>54.8±13.9	0.014 [0.011 - 0.028]<br>0.020±0.016	0.001 [0.0006 - 0.0022]<br>0.001±0.0009

Data are presented as median [IQR] mean±SD

Supplemental Table 1. Increased percentage of Endothelial progenitor and T_{ang} cells in SLE patients.
The descriptive statistics of each distribution reported in Figure 2 is shown in the table.

	% $CD28^{null}$ in $CD3^+$	% $CD28^{null}$ in $CD3^+CD4^+$	% $CD28^{null}$ in $CD3^+CD8^+$	% $CD28^{null}$ in Tang $CD3^+$	% $CD28^{null}$ in Tang $CD3^+CD4^+$	% $CD28^{null}$ in Tang $CD3^+CD8^+$
**HC**	10.7 [3.5 - 17.1]<br>11.6±5.9	0.9 [0.2 - 2.4]<br>2.3±3.8	25.1 [14.2 - 37.7]<br>26.2±11.6	7.6 [2.8 - 11.6]<br>8.0±4.0	0.8 [0.2 - 1.5]<br>0.9±0.8	11.5 [7.8 – 24.0]<br>14.9±8.3
**SLE**	21.8 [10.47 - 36.6]<br>25.1±15.9	6.4 [1.2 - 12.1]<br>10.0±13.3	45.5 [20.1 - 59.9]<br>43.9±19.6	11.2 [8.5 - 21.6]<br>15.6±11.0	1.2 [0.5 - 2.6]<br>3.1±6.8	24.4 [13.2 – 34.2]<br>25.0±13.6

Data are presented as median [IQR] mean±SD

Supplemental Table 2. Senescent angiogenic T cells characterize SLE patients.
The descriptive statistics of each distribution reported in Figure 3 is shown in the table.

	SLEDAI-2K	ESR	CRP	Chol. [TOT]	Chol. [LDL]
**% Tang CD3$^+$CD4$^+$**	**x** = 28.5 [24.3 - 39.8]<br>31.6±9.9<br>**y** = 3.0 [2.0 - 4.0]<br>3.4±2.6	--	--	--	--
**% early EPC**	--	**x** = 0.01 [0.01 - 0.02]<br>0.02±0.01<br>**y** = 10.0 [5.0 - 16.0]<br>11.5±8.3	**x** = 0.01 [0.01 - 0.02]<br>0.02±0.01<br>**y** = 0.2 [0.1 - 0.5]<br>0.5±1.0	--	--
**% late EPC**	--	**x** = 0.001 [0.006 - 0.002]<br>0.001±0.0009<br>**y** = 10.0 [5.0 - 16.0]<br>11.5±8.3	--	--	--
**% CD28^{null} in Tang CD3$^+$**	--	--	--	**x** = 10.9 [8.1 - 20.4]<br>15.1±11.1<br>**y** = 168.0 [138 - 184]<br>165.8±28.9	**x** = 10.5 [6.9 - 20.4]<br>15.1±12.4<br>**y** = 88.0 [74.0 - 108.0]<br>86.1±27.6
**% CD28^{null} in CD3$^+$CD4$^+$**	--	--	--	**x** = 5.4 [1.0 - 12.4]<br>10.0±13.7<br>**y** = 168.0 [138.0 - 184.0]<br>165.8±28.9	**x** = 3.1 [0.7 - 12.4]<br>9.5±14.7<br>**y** = 88.0 [74.0 - 108.0]<br>86.1±27.6

Data are presented as median [IQR] mean±SD

Supplemental Table 3. Impaired percentage of circulating EPCs and T_{ang} is disease activity related.
The descriptive statistics of each distribution reported in Figure 4 is shown in the table.

	IL-8 of Low % Tang CD3	IL-8 of High % Tang CD3	IL-8	MMP-9
	47.0 [30.5 - 69.5] 65.2±65.5	263.0 [135.8 - 1195] 576.5±577.4	--	--
**% Tang $CD3^+$**	--	--	**x** = 42.2 [37.6 - 49.6] 43.0±8.9 **y** = 158 [45.2 - 382.5] 323±415.6	**x** = 42.2 [37.6 - 49.6] 43.0±8.9 **y** = 495.0 [312.5 - 721.5] 546.6±309.1
**% Tang $CD3^+CD4^+$**	--	--	**x** = 33.2 [26.3 - 42.6] 34.3±9.8 **y** = 158 [45.2 - 382.5] 323±415.6	--
**% CD28nul in Tang $CD3^+$**	--	--	**x** = 10.5 [6.8 - 16.28] 13.0±9.9 **y** = 158 [45.2 - 382.5] 323±415.6	--
**% $CD28^{null}$ in Tang $CD3^+CD4^+$**	--	--	**x** = 1.23 [0.5 - 2.1] 2.4±5.6 **y** = 158 [45.2 - 382.5] 323±415.6	--
**% CD28nul in Tang $CD3^+CD8^+$**	--	--	**x** = 18.35 [10.9 - 30.4] 21.7±12.9 **y** = 158 [45.2 - 382.5] 323±415.6	--
**% $CD34^+$**	--	--	--	**x** = 0.05 [0.04 - 0.09] 0.06±0.03 **y** = 493 [309.0 - 706.0] 519.9±277.2

Data are presented as median [IQR] mean±SD

Supplemental Table 4. Circulating proangiogenic factors reflect angiogenic circulating cells related compartments. The descriptive statistics of each distribution reported in Figure 5 is shown in the table.

	<33th percentile		>66th percentile	
	HCs	SLE	HCs	SLE
% Tang CD3	56.0 [51.3 - 56.1] 54.4±2.7	54.4 [49.9 – 55.0] 52.8±3.3	36.5 [34.9 – 43.1] 38.1±4.3	34.3 [25.6 – 75.3] 31.8±6.3
% Tang $CD3^+CD4^+$	48.8 [43.4 – 49.3] 47.1±3.2	43.3 [36.4 – 49.7] 43.1±7.3	**31.8 [26.8 – 32.4]*§ 30.3±3.0**	**25.5 [21.0 – 26.6]*§ 23.0±4.9**
% Tang $CD3^+CD8^+$	69.8 [65.3 – 73.8] 69.6±4.2	73.2 [55.5 – 78.9] 68.4±13.9	52.9 [47.2 – 55.6] 51.9±4.2	46.7 [34.9 – 57.1] 46.8±10.8
% $CD28^{null}$ in Tang $CD3^+$	5.2 [4.4 – 8.7] 6.1±2.2	8.1 [6.2 – 10.7] 8.3±2.3	11.1 [7.8 – 15.4] 11.4±3.8	14.5 [6.9 – 28.3] 20.3±16.4
% $CD28^{null}$ in Tang $CD3^+CD4^+$	0.2 [0.2 – 2.3] 0.9±1.2	0.5 [0.3 – 1.6] 0.8±0.7	1.3 [1.3 – 2.1] 1.6±0.4	1.7 [0.6 – 4.3] 6.1±11.2
% $CD28^{null}$ in Tang $CD3^+CD8^+$	9.2 [8.0 – 16.1] 11.1±4.3	13.5 [9.5 – 21.7] 15.2±6.6	25.2 [12.1 – 29.6] 22.3±9.1	31.2 [13.2 – 40.4] 31.2±17.4
% $CD28^{null}$ in CD3	9.5 [5.7 – 12.0] 9.0±3.1	7.5 [6.7 – 24.6] 14.0±11.7	16.0 [12.6 – 20.6] 16.4±4.0	24.1 [14.9 – 42.0] 29.1±20.7
% $CD28^{null}$ in $CD3^+CD4^+$	0.2 [0.2 – 1.9] 0.7±0.9	0.7 [0.2 – 4.2] 1.9±2.2	4.1 [1.4 – 12.8] 6.1±5.9	8.1 [1.9 – 26.4] 15.8±18.7
% $CD28^{null}$ in $CD3^+CD8^+$	22.0 [13.9 – 28.2] 21.3±7.1	17.3 [14.6 - 48.1] 28.5±18.2	36.4 [29.8 – 41.9] 36.0±6.0	45.8 [44.1 – 66.0] 50.4±19.6

Data are presented as median [IQR] mean±SD

Supplemental Table 5. Percentage of Tang subsets of SLE patients and HCs stratified by the tertile of the percentage of circulating T_{ang} $CD3^+$ cells (low, <33th percentile; high, >66th percentile).
§ Statistical analysis of the differences among HCs vs SLE was performed by Mann-Whitney test. p values <0.05 were considered significant: * $p<0.05$.

	SLE	HCs	p-value
**IL-6, pg/ml**	2.30 [2.30-5.95]	9.25 [4.37-20.13]	**0.01***
**IL-8, pg/ml**	66.00 34.75-192.80]	607.5 [206.5-1090]	**0.001****
**MMP-9, ng/ml**	441.0 [259.8-531.0]	706.0 [510.3-802.0]	**0.007****
**IFN-γ, pg/ml**	13.0 [13.0-14.75]	13.0 [13.50-15.0]	0.784

Data are presented as median [IQR] mean±SD

Supplemental Table 6. IL-6, IL-8, MMP-9 and IFN-γ serum levels from patients and controls.
Statistical analysis of the differences was performed by Mann-Whitney test. p values <0.05 were considered significant: * p<0.05, ** p<0.001.
List of abbreviations: HCs, healthy controls; IFN, interferon; IL, interleukin; IQR, interquartile range; MMP, matrix metalloproteinase; SLE, systemic lupus erythematosus.

	IL-6 mRNA	IL-6 protein expression	ICAM-1 mRNA	ELAM-1 mRNA	MMP-9 protein expression
**Low % Tang CD3**	3.14 [2.19 - 4.8]<br>3.3±1.4	0.3 [0.07 - 0.6]<br>0.5±0.6	2.0 [1.3 - 2.1]<br>1.8±0.4	2.0 [1.1 - 3.2]<br>2.1±1.0	
**High % Tang CD3**	1.6 [1.0 - 1.8]<br>1.4±0.4	0.1 [0.0 - 0.1]<br>0.07±0.07	0.7 [0.4 - 0.9]<br>0.7±0.2	0.5 [0.3 - 0.8]<br>0.5±0.2	
**% Tang $CD3^+$**					**x** = 44.7 [33.2 - 54.3]<br>43.1±12.3<br>**y** = 1.4 [0.6 - 3.8]<br>2.4±2.7
**% $CD28^{null}$ in Tang $CD3^+$**					**x** = 9.3 [5.2 - 23.6]<br>15.6±15.2<br>**y** = 1.4 [0.6 - 3.8]<br>2.4±2.7
**% $CD28^{null}$ in Tang $CD3^+CD4^+$**					**x** = 1.1 [0.2 - 3.2]<br>4.3±9.6<br>**y** = 1.4 [0.6 - 3.8]<br>2.4±2.7
**% $CD28^{null}$ in Tang $CD3^+CD8^+$**					**x** = 25.1 [9.3 - 36.2]<br>25.2±18.3<br>**y** = 1.4 [0.6 - 3.8]<br>2.4±2.7

Data are presented as median [IQR] mean±SD

Supplemental Table 7. Low percentage of circulating T_{ang} cells induces pro-inflammatory endothelial phenotype. Correlation between the percentage of cell subpopulations and the expression of factors at mRNA and protein level, after HUVECs' stimulation with the sera from patients and controls.
The descriptive statistics of each distribution reported in Figure 6 is shown in the table.

	SLE	HCs	p-value
**IL-6, fold change [RQ]**	1.91 [1.27-3.14]	1.82 [1.80-3.50]	0.904
**MMP-9, fold change [RQ]**	0.81 [0.36-1.20]	1.12 [1.10-1.22]	0.143
**IFN-γ, fold change [RQ]**	N.D.	N.D.	N.P.
**ICAM-1, fold change [RQ]**	1.00 [0.60-2.15]	0.70 [0.60-0.80]	0.476
**ELAM, fold change [RQ]**	0.90 [0.35-2.90]	0.70 [0.60-0.80]	0.667

Data are presented as median [IQR] mean±SD

Supplemental Table 8. IL-6, MMP-9, IFN-γ, ICAM-1 and ELAM-1 mRNA expression levels in HUVECs treated with serum from patients and controls.
Statistical analysis of the differences was performed by Mann-Whitney test.
List of abbreviations: ELAM, endothelial cell leukocyte adhesion molecule; HCs, healthy controls; ICAM, inter-cellular adhesion molecule; IFN, interferon; IL, interleukin; MMP, matrix metalloproteinase; N.D., not detectable; N.P., not performed;SLE, systemic lupus erythematosus.

Relative protein level	SLE	HCs	p-value
**IL-6**	0.10<br>[0.0-0.30]<br>0.12±0.13	0.45<br>[0.12-1.67]<br>0.75±0.87	0.079
**IL-8**	N.D.	N.D.	N.P.
**MMP-9**	1.20<br>[0.70-1.80]<br>1.36±0.90	3.30<br>[1.72-8.02]<br>4.35±3.48	**0.034***
**IFN-γ**	0.85<br>[0.50-1.05]<br>0.85±0.38	0.55<br>[0.50-1.12]<br>0.73±0.39	0.606

Data are presented as median [IQR] mean±SD

Supplemental Table 9. IL-6, IL-8 and MMP-9 protein expression levels in HUVECs treated with sera from patients and controls.
Statistical analysis of the differences was performed by Mann-Whitney test. p values <0.05 were considered significant: * p<0.05
List of abbreviations: HCs: healthy controls; IFN: interferon; IL: interleukin; IQR: interquartile range; MMP: matrix metalloproteinase; N.D.: not detectable; N.P.: not performed; SLE: systemic lupus erythematosus.

	IL-8	% early EPC
**% proliferation**	**x** = 128.0 [115.0 - 168.5]<br>142.9±42.4<br>**y** = 118.0 [42.0 - 273.0]<br>277.4±393.1	**x** = 128.0 [115.0 - 168.5]<br>142.9±42.4<br>**y** = 0.01 [0.008 - 0.02]<br>0.02±0.01

Data are presented as median [IQR] mean±SD

Supplemental Table 10. Serum IL-8 correlates with endothelial progenitor cells and HUVECs proliferation. The descriptive statistics of each distribution reported in Figure 7 is shown in the table.

	SLE	HCs	p-value
**MTT 24 h, %**	62.0 [28.0-75.0]	81.0 [61.50-86.0]	0.189
**MTT 72 h, %**	117.0 [115.0-152.0]	158.0 [114.8-193.0]	0.479

Data are presented as median [IQR] mean±SD

Supplemental Table 11. Proliferation rate of HUVECs treated with sera from patients and controls.
Statistical analysis of the differences was performed by Mann-Whitney test.
List of abbreviations: HCs: healthy controls; h: hours; SLE: systemic lupus erythematosus.
